# Feasibility of an implantable bioreactor for renal cell therapy using silicon nanopore membranes

**DOI:** 10.1038/s41467-023-39888-2

**Published:** 2023-08-29

**Authors:** Eun Jung Kim, Caressa Chen, Rebecca Gologorsky, Ana Santandreu, Alonso Torres, Nathan Wright, Mark S. Goodin, Jarrett Moyer, Benjamin W. Chui, Charles Blaha, Paul Brakeman, Shant Vartanian, Qizhi Tang, H. David Humes, William H. Fissell, Shuvo Roy

**Affiliations:** 1 University of California, San Francisco, CA USA; 2 Silicon Kidney LLC, San Ramon, CA USA; 3 SimuTech Group, Rochester, NY USA; 4 University of Michigan, Ann Arbor, MI USA; 5 Innovative Biotherapies Inc, Ann Arbor, MI USA; 6 Vanderbilt University Medical Center, Nashville, TN USA

**Keywords:** Nanopores, Renal replacement therapy, Biomimetics, Biomedical engineering

## Abstract

The definitive treatment for end-stage renal disease is kidney transplantation, which remains limited by organ availability and post-transplant complications. Alternatively, an implantable bioartificial kidney could address both problems while enhancing the quality and length of patient life. An implantable bioartificial kidney requires a bioreactor containing renal cells to replicate key native cell functions, such as water and solute reabsorption, and metabolic and endocrinologic functions. Here, we report a proof-of-concept implantable bioreactor containing silicon nanopore membranes to offer a level of immunoprotection to human renal epithelial cells. After implantation into pigs without systemic anticoagulation or immunosuppression therapy for 7 days, we show that cells maintain >90% viability and functionality, with normal or elevated transporter gene expression and vitamin D activation. Despite implantation into a xenograft model, we find that cells exhibit minimal damage, and recipient cytokine levels are not suggestive of hyperacute rejection. These initial data confirm the potential feasibility of an implantable bioreactor for renal cell therapy utilizing silicon nanopore membranes.

## Abstract (web summary)

An implantable bioartificial kidney with a cell-containing bioreactor could be used to treat end-stage renal disease. Here the authors demonstrate the feasibility of an implantable bioreactor by maintaining human cell viability and functionality after implantation in a xenograft model.

## Introduction

More than 2 million people worldwide receive treatment for end-stage renal disease (ESRD), a devastating diagnosis with increasing incidence due to escalating rates of diabetes mellitus and hypertension^1–3^. While kidney transplantation offers excellent outcomes, with a 5-year survival of >80% for recipients of living donor organs, lifelong immunosuppression carries serious health complications, and graft rejection is of perpetual concern^4–6^. Furthermore, the demand for donor organs far exceeds supply; just over 20,000 kidney transplants are performed annually in the U.S., and less than 20% of those waitlisted for an organ ever receive one^1,7^. Thus, nearly half a million U.S. ESRD patients are dependent on thrice weekly multi-hour sessions of hemodialysis—a morbid and life-limiting therapy with almost 60% mortality at 5 years^1^. Hemodialysis only partially replaces native renal function, and most dialysis patients still experience symptoms and sequelae of ESRD, including endocrine and cognitive dysfunction^8–10^.

The limitations of dialysis treatment on patient health and quality of life have led to a growing interest in alternative renal replacement therapies^11^. Current approaches utilize cell-based strategies to create a fully functional replacement organ^12–16^, wearable artificial kidney dialysis devices that provide prolonged treatment outside of the clinical setting^17–19^, or biohybrid devices that use both cells and artificial materials to recapitulate functions of the tubule and glomerulus, respectively^20–23^. Previous success has been reported using an extracorporeal renal tubule assist device (RAD) containing human renal epithelial cells (HREC). In a phase II clinical trial, the addition of the RAD to renal replacement therapy improved patient survival, reducing the risk of death by 50%^24^.

Our group has adopted the biohybrid approach of the RAD to develop a completely implantable bioartificial kidney (iBAK); to mimic human nephron physiology, we envision an iBAK composed of a silicon hemofilter to recapitulate the selective permeability of the glomerulus, coupled with an immunoprotective bioreactor containing renal tubule cells to reabsorb solutes and water and recreate the metabolic and endocrinologic function of the renal tubule^25,26^. In the proposed iBAK, HREC are encapsulated within the bioreactor to continuously process the ultrafiltrate from the hemofilter and selectively return sodium ions and water back to the circulatory system to ensure volume homeostasis. The toxins and excess solutes are concentrated in the remaining water and directed to the bladder for excretion. Using semiconductor microfabrication techniques, silicon wafers are precisely engineered to reproducibly create thin (<1 μm) biomimetic membranes that incorporate monodispersed nanoscale slit pores^27,28^. These silicon nanopore membranes (SNM) are uniquely suited to serve as an exclusion barrier due to their high molecular selectivity^29,30^. Moreover, their high mass transfer characteristics supplemented with surface modification strategies to enhance biocompatibility enable “pumpless” blood filtration solely using innate cardiac perfusion pressure^27,29^. Previously, we described the fabrication and testing of implantable hemofilters constructed from SNM to simulate glomerular ultrafiltration^31^. These prototype hemofilters successfully demonstrated continuous ultrafiltration in vivo under the innate recipient arteriovenous pressure differential in mongrel dogs treated with only antiplatelet agents^31^.

Here, we focus on the bioreactor component of the iBAK and describe the first steps in the development of an implantable device to replicate key functions of the renal tubule, which is intended to ultimately function in tandem with an implantable hemofilter. To demonstrate the possible feasibility of an implantable bioreactor, we decided to first investigate the capacity of the SNM to sustain primary HREC in vivo using only antiplatelet agents and without immunosuppression. To this end, a prototype bioreactor design was optimized for blood flow and constructed to house SNM with 10-nm-wide pores and HREC cultured on acrylic cell inserts. Device prototypes were surgically xenotransplanted into pigs for up to 7 days. The SNM pores allowed the passage of nutrients, rendering the encapsulated cells viable and functional. These results demonstrate the possibility of using an SNM-based bioreactor that could be integrated into a future iBAK that could potentially operate without recipient immunosuppression.

## Results

### Silicon nanopore membranes (SNM) protect human renal epithelial cells (HREC) from immune mediators in vitro, ensuring cell viability and function

**Fig. 1:**
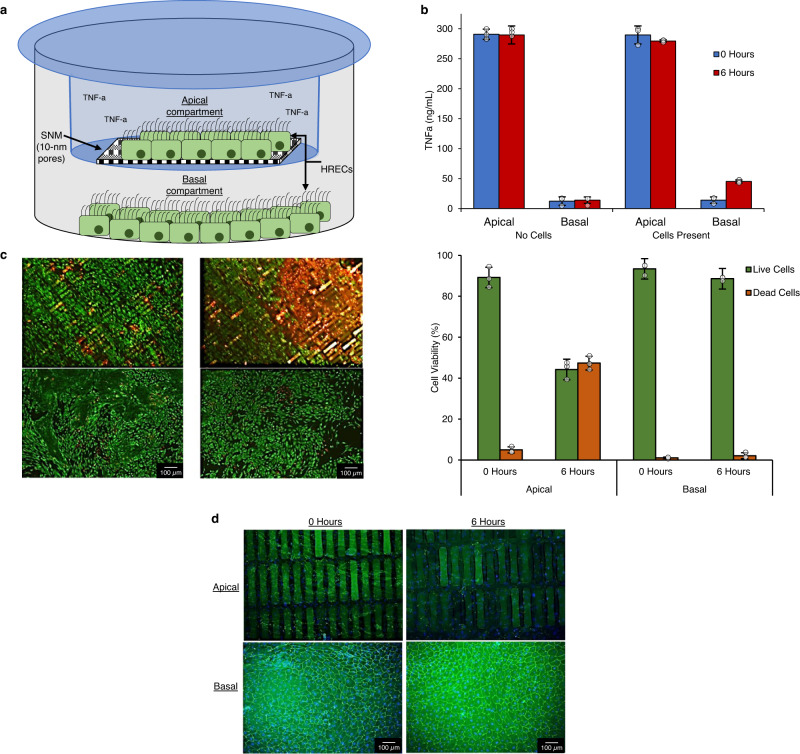
Silicon nanopore membranes (SNM) prevent diffusion of cytokines in vitro. **a** In vitro setup where human renal epithelial cells (HRECs) were cultured in apical and basal compartments of a 6-well plate, separated by an SNM bonded to a Transwell® membrane insert (blue). TNF-alpha (TNF-a) (300 ng/mL) was added to the apical compartment only and incubated for 6 h. **b** TNF-a concentration in the apical and basal compartments after 6 h, with and without HREC. There was no statistically significant change in TNF-a concentration within the apical compartment after 6 h [290.81 ± 8.51 and 289.72 ± 15.01 (mean ± standard deviation)]. The TNF-a concentration on the basal side of the SNM was 12.38 ± 7.05 (mean ± standard deviation) after 6 h, suggesting little to no passage of TNF-a through the SNM. In the presence of cells, basal concentration after 6 h was 45.16 ± 2.57 (mean ± standard deviation) (*n* = 3 independent experiments). **c** LIVE/DEAD™ imaging of apical and basal HREC using calcein-AM (green) and ethidium homodimer-1 (red) demonstrated more dead cells in the apical compartment after 6 h, relative to the 0-h time point and relative to the basal compartment. Cells exposed to TNF-a in the apical compartment demonstrated 44.2 ± 4.3% viability (mean ± standard deviation) after 6 h. Cells on the basal side of the SNM demonstrated 88.6 ± 2.1% viability (mean ± standard deviation) after 6 h (*n* = 3 independent experiments). **d** Tight junction and cell monolayer integrity were evaluated by ZO-1 expression (green) and DAPI nuclear staining (blue). Cells on the basal compartment maintained ZO-1 expression after 6 h. Cells directly exposed to TNF-a in the apical compartment had fewer cells (indicated by DAPI) with less defined ZO-1 expression relative to cells at 0 h. Note: apical cells were cultured directly on SNM, which attenuated immunofluorescence imaging. Nonetheless, the difference in DAPI and ZO-1 expression between 0 and 6-h time points remains evident (*n* = 3 independent experiments). Source data are provided as a Source Data file.

In a functional bioreactor, HREC must be housed such that they receive oxygen and nutrients but are protected from recipient immune cells. To confirm whether the SNM could provide a potentially immunoprotective barrier in vitro, we exposed one side of an SNM to a supraphysiological concentration of the pro-inflammatory cytokine, tumor necrosis factor-α (TNF-a). Two monolayers of HRECs were separated by an SNM bonded to a Transwell® membrane suspended in a 6-well tissue culture plate. The cell monolayer on the apical side of the SNM (“apical compartment”) was grown to confluency; similarly, the cell monolayer on the basal side of the SNM (“basal compartment”) was grown to confluency on the tissue culture plate. TNF-a in cell media was added to the apical compartment only and incubated for 6 h (Fig. 1a).

Six hours after adding TNF-a-containing cell media to the apical compartment, TNF-a levels in the apical compartment remained close to the starting concentration (300 ng/mL), while TNF-a levels in the basal compartment remained negligible. Despite the presence of a cell monolayer on the apical side, the SNM with 10-nm-wide pores blocked the passage of TNF-a to the basal side (Fig. 1b). Similarly, in a control setup without cells seeded onto the SNM, the TNF-a concentrations in both the apical and basal compartments did not change after 6 h, demonstrating that the SNM was responsible for limiting TNF-a diffusion.

After TNF-a exposure, the viability of both HREC monolayers was quantified with the LIVE/DEAD™ assay. All cells in the apical compartment, those directly exposed to TNF-a, exhibited cell death with less than 50% cell viability. Cells in the basal compartment maintained high rates of viability (approaching 90%) (Fig. 1c).

Expression of Zonula Occludens-1 (ZO-1), a tight junction protein located on the cytoplasmic surface, and cell nuclei were visualized using immunofluorescence staining. After TNF-a exposure, ZO-1 expression was lower in HREC in the apical compartment, relative to initial apical cell ZO-1 expression and to basal compartment cell expression. In the absence of TNF-a, HREC from the basal compartment showed consistent and regular ZO-1 staining at intercellular junctions; localization and expression levels of ZO-1 in basal cells were similar at time points 0 and 6 h (Fig. 1d).

### Thrombosis and protein fouling can be minimized in a hemocompatible flow path of an implantable intravascular bioreactor containing HREC

**Fig. 2:**
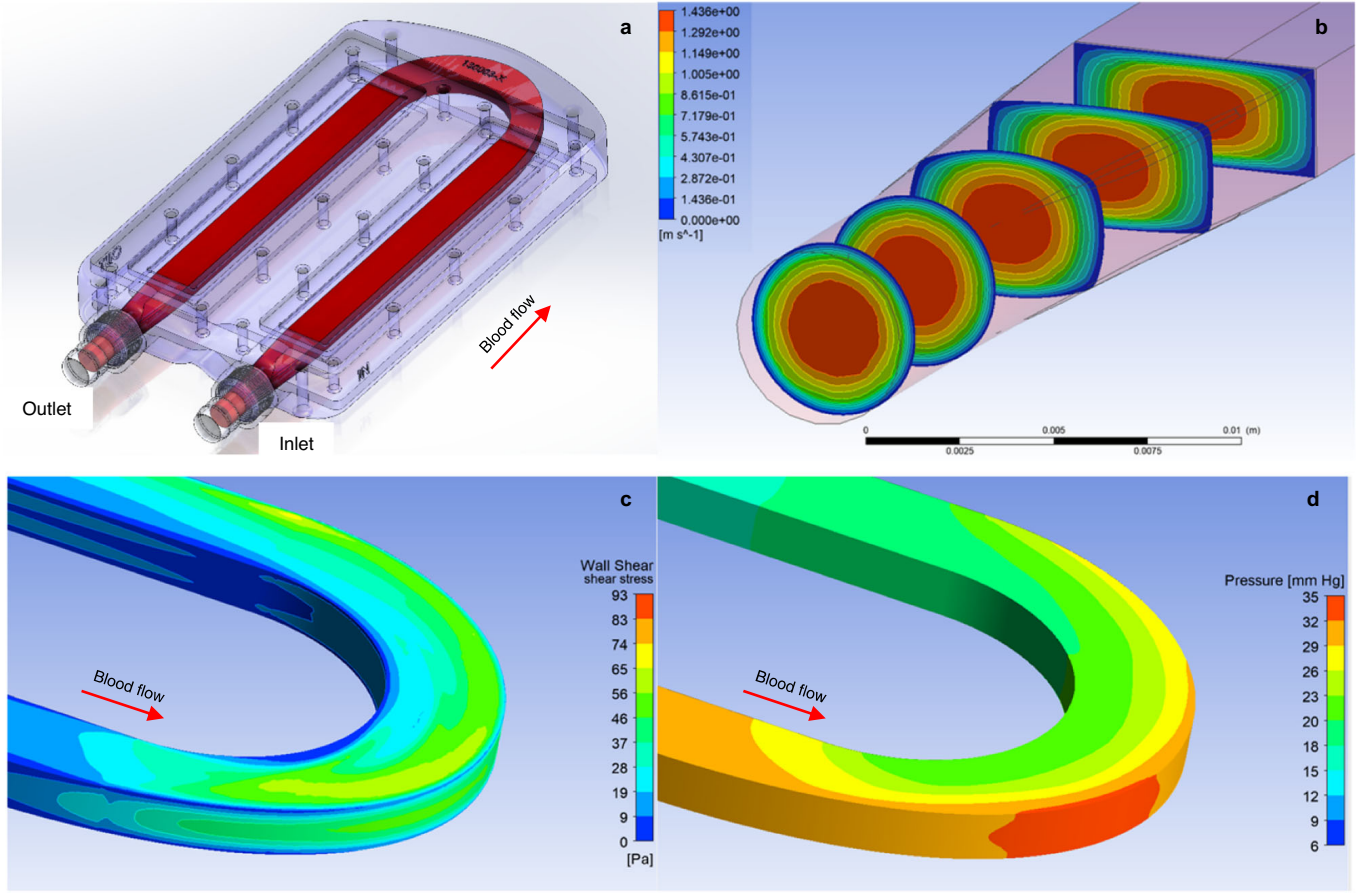
In silico optimization of the blood flow path. Computational fluid dynamics (CFD) modeling was performed iteratively to optimize the geometry of the blood flow path, with special considerations made for the U-turn which connects the blood inlet and outlet ports (**a**) and the transition from the circular blood inlet/outlet shape to the rectangular cross-section of the parallel-plate arrangement of the SNM (**b**). A time-varying mass flow rate inlet boundary condition previously derived from animal data was utilized. The conduit geometry was modified until the wall shear stress was noted to be above the lower threshold of 1 Pa to reduce the risk of thrombosis formation but stayed well below the higher ranges associated with hemolysis (**c**). A low pressure drop of 20–35 mmHg was predicted for the U-turn flow path (**d**).

Low wall shear stress (WSS) regions were minimized by in silico optimization of blood conduit geometry (Fig. 2). To eliminate flow-induced thrombogenesis inside the bioreactor, we used computational fluid dynamics (CFD) to optimize pulsatile blood flow fields with two primary design criteria. First, the cross-sectional shape of the blood path needed to transition from the circular conduits of vasculature and connecting vascular grafts to a rectangular duct flow defined by a parallel-plate arrangement of SNM and then back to vasculature (Fig. 2a, b). Second, to facilitate surgical anastomosis to an artery and vein of similar size, a hilum-like configuration was designed with inflow and outflow conduits antiparallel and adjacent to each other. These conditions were further constrained by the need to avoid stagnant or recirculating flow to prevent thrombosis. By iterative design modification and CFD analysis, we arrived at a 3-mm-high U-shaped blood flow path that satisfied these criteria (Fig. 2a). The inlet and outlet ports were separated by 4 cm. The blood flow field was laminar and maintained WSS greater than 1 Pa for flow rates of 1000–1750 mL/min and pressure drop of 20–35 mmHg (Fig. 2c, d).

**Fig. 3:**
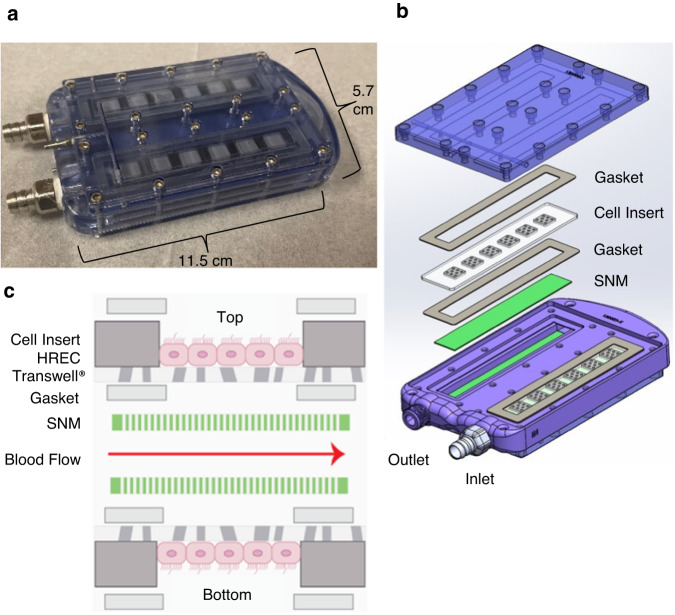
Bioreactor design with constituent components. **a** A fully assembled bioreactor prior to implant, with dimensions of 11.5 × 5.7 × 1.8 cm (length × width × height). **b** Detailed layering of bioreactor components, with one section composed of an SNM, and an acrylic cell insert, sandwiched by two silicone gaskets. A complete bioreactor contains four sections for a total of four SNM, four cell inserts, and eight silicone gaskets. **c** Blood flow channels are in direct contact with SNM, which are separated from the cell insert by a silicone gasket (cross-sectional view shown). The cell insert is constructed by epoxying a Transwell® membrane to an acrylic piece, which is subsequently seeded with HREC.

HRECs were encapsulated in an implantable bioreactor containing biocompatible SNM (Fig. 3). Bioreactor prototypes were assembled under sterile conditions from polycarbonate machined parts with an internal U-shaped blood flow conduit, stainless steel connectors, silicone gaskets, acrylic cell inserts, and SNM (Fig. 3a). The SNM were coated with 1–2 nm-thick polyethylene glycol (PEG) films deposited by self-assembly techniques that did not occlude the pores^28^. Each bioreactor consisted of four SNM within the polycarbonate housing in-line with the top and bottom surfaces of the blood channel. On the opposite side of the SNM were the acrylic cell inserts with confluent HREC (Fig. 3b, c). The basal side of HREC was in direct contact, allowing for exchange with the ultrafiltrate produced by the SNM. While the ultrafiltrate in the apical space was not collected in this study, we expect future studies to siphon the filtrate to the urinary bladder or to a transcutaneous collection port for subsequent analysis. After sealing the cell inserts and SNM with silicone gaskets, stainless steel connectors were attached to the polycarbonate body to transition the blood between 6 mm-diameter ringed polytetrafluoroethylene (PTFE) vascular grafts and the 3 mm-high parallel-plate arrangements of SNM. The 10 nm-wide pores in the SNM rendered the SNM-based portions of the blood channel walls permeable to nutrients and waste exchange between the blood flow path and encapsulated cells. After assembly, the device was filled with cell culture media, inlets and outlets were capped, and the device was placed in a sterile sleeve until implantation.

### A bioreactor containing SNM and HREC was successfully implanted in pigs and remained patent, functional, and thrombus-free without immunosuppression or therapeutic systemic anticoagulation for up to 7 days

No adverse animal responses were observed during the 3- and 7-day implantations. To evaluate the biocompatibility of the device, bioreactors containing HRECs were implanted into healthy adolescent Yucatan pigs on dual antiplatelet therapy. Bioreactors were connected through vascular PTFE grafts to the carotid artery and jugular vein or iliac artery and iliac vein. Post-operatively there were no signs of complications related to the devices or the surgeries, including bleeding, infection, or skin breakdown. The animals remained in good health for the duration of the experiment, and no indications of hyperacute rejection were noted. In total, five implants were performed successfully.

**Fig. 4:**
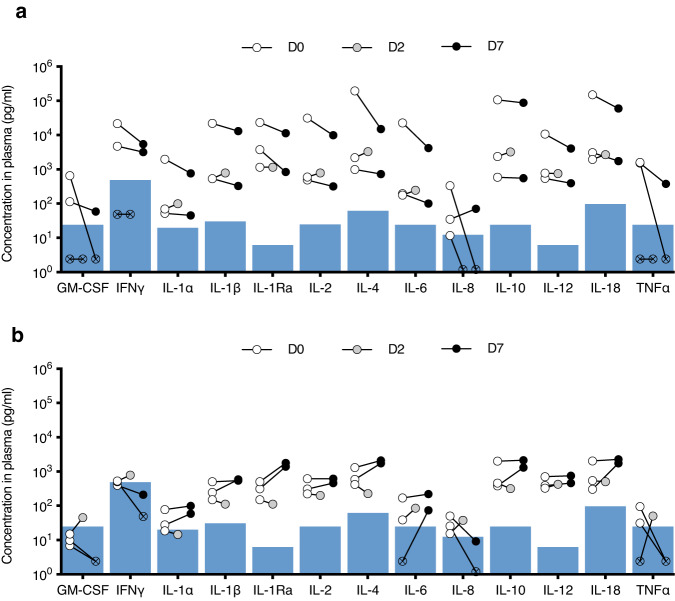
Recipient pig plasma cytokine concentration before and after device implantation. The concentrations of 13 porcine cytokines in the recipient plasma on days 0 (time of implant), 2, and 7 after implantation of bioreactor devices (**a**) and control devices that did not contain cells (**b**) (*n* = 3 each type of device). Each circle represents the average concentration (*n* = 3 plasma samples) of one animal, with connected symbols representing measurements from the same animal. The lower limit of the linear range of detection (LLD) for each analyte is represented by the blue-shaded area in both groups. Symbols with X are not detected but arbitrarily given values that are 1/10 of the LLD to be plotted on the logarithmic scale. Overall, bioreactor recipients demonstrated decreased cytokine expression by day 7, reflecting a state of minimal inflammation, as opposed to a robust immune response characteristic of acute rejection. Similarly, cytokine expression in devices without cells was low and did not markedly change. Source data are provided as a Source Data file.

Recipient cytokine levels were not persistently elevated after 7 days of implantation. We monitored recipient cytokine expression to assess if the implantation of the devices with or without xenogeneic human cells provoked immune activation. Pig plasma was collected on the day of implantation at the end of the procedure (day 0) and on days 2 and 7 after implantation, and plasma cytokine concentrations were quantified. Cytokine concentrations on day 0 of cell-containing devices were elevated compared to normal human plasma levels^32^, likely due to the acute response to the surgical procedure (Fig. 4a). Concentrations of all cytokines remained the same or slightly elevated on day 2 and decreased on day 7. By comparison, cytokine concentrations in the recipients of devices without cells were relatively low and did not change markedly on days 2 and 7 (Fig. 4b).

**Fig. 5:**
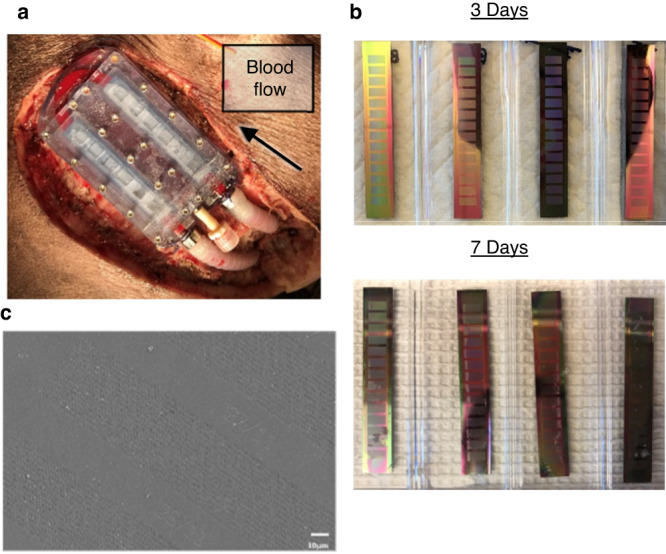
Explanted devices did not demonstrate evidence of thrombosis. **a** The bioreactor in situ at the time of explant, with blood flow demonstrating patency in the arterial and venous grafts and the device. (Note: no DragonSkin silicone shown). **b** SNM after explant at days 3 or 7 demonstrated no gross thrombi formation. **c** Scanning electron microscopy (SEM) image of representative SNM surface showed minimal cellular debris after implant into an immunocompetent pig (500× magnification) (*n* = 20 SNM from 5 animals).

The blood flow path was patent, and both the bioreactor and SNM were intact upon explant. Device and vascular graft patency were confirmed grossly (Fig. 5a) and with an angiogram on the day of the explant. After 3 or 7 days in vivo, the bioreactor was removed from the animal and disassembled for evaluation. Gross examination revealed that the SNM was intact with punctate air bubbles in the outward-facing cell chambers. There was no gross evidence of thrombus formation within the device or on the SNM, indicating the absence of significant blood stagnation (Fig. 5b). Scanning electron microscopy (SEM) of the SNM blood-contacting surfaces showed minimal presence of remnants of blood cells and platelets (Fig. 5c).

**Fig. 6:**
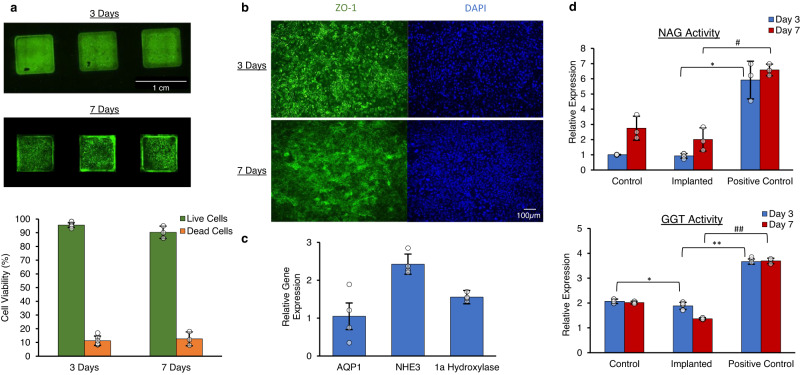
Analyses of implanted cells. **a** Immunofluorescence imaging using LIVE/DEAD™ staining demonstrated high cell viability on cell inserts after 3- and 7-day implants. Quantification of LIVE/DEAD™ staining demonstrated 95.6 ± 1.7 and 90.3 ± 4.5% viability (mean ± standard deviation) of cell inserts after 3- and 7-day implants, respectively (for 3-day implants *n* = 6 biologically independent samples from 3 animals, for 7-day implants *n* = 3 independent samples from 2 animals). **b** Cell inserts after 3- and 7-day implants demonstrated confluency and integrity of the cell monolayer, as examined by immunofluorescence imaging of tight junction protein ZO-1 (green) and DAPI (blue) nuclear staining (for 3-day implants *n* = 9, for 7-day implants *n* = 6). **c** Gene expression of cell transporters AQP1 and NHE3 and vitamin D 1a Hydroxylase were measured using qPCR as a surrogate of renal cell function. Relative to in vitro controls, AQP1 expression was 1.05x ± 0.35 (mean ± standard deviation), while 1a Hydroxylase and NHE3 were 1.56 ± 0.18 and 2.43 ± 0.27 x more expressed (*n* = 4 biologically independent samples). **d** The activity of damage biomarkers NAG and GGT was measured after days 3 and 7, using ELISA and normalized to in vitro controls. HREC from implants demonstrated NAG expression levels similar to those of in vitro controls and were significantly lower than a standard positive control (0.93 ± 0.15 for day 3) (2.01 ± 0.77 for day 7) (mean values ± standard deviation). Day 7 GGT levels were comparable to in vitro control cells and significantly lower than a standard positive control (1.37 ± 0.04) (*n* = 3 biologically independent samples). * or # *p* < 0.05, ** or ## *p* < 0.001, where * is for day 3 comparisons and # is for day 7. Source data are provided as a Source Data file.

After both 3 and 7 days, the encapsulated cells maintained >90% cell viability relative to in vitro controls (Fig. 6a). There was no evidence of cell detachment, and confluence was maintained. The integrity of the HREC monolayer, as examined by ZO-1 protein expression, indicated the maintenance of intercellular tight junctions. Cells encapsulated within the bioreactor demonstrated ZO-1 expression comparable to in vitro control cells (Fig. 6b).

Encapsulated cells had normal or elevated expression of proximal tubule cell-specific functional markers. Gene expression of HREC harvested from the explanted bioreactor was measured using qPCR. HREC-specific markers were selected as surrogates of functionality and compared to in vitro control cells. Expression of sodium-hydrogen exchanger 3 (NHE3), a transporter on the apical cell surface responsible for sodium balance and, indirectly, blood pH, and 25-hydroxyvitamin D3 1-∝-hydroxylase (1a Hydroxylase), which catalyzes the hydroxylation of vitamin D and maintains calcium homeostasis, were both significantly elevated in the encapsulated cells. The expression of aquaporin 1 (AQP1), a molecular water channel protein present on apical and basal surfaces of renal cells, was comparable to in vitro control cells after 3 days (Fig. 6c).

Kidney injury biomarker levels were not upregulated in encapsulated HREC implanted into pigs. HREC encapsulated in the bioreactor exhibited low levels of *N*-acetyl-β-d-glucosaminidase (NAG) activity, a sensitive marker of kidney cell damage. NAG activity in the encapsulated cells was comparable to in vitro control cells and less than 10% of standard positive control. The activity levels of gamma-glutamyl transpeptidase (GGT), a biomarker for ischemic injury as well as graft rejection, were also comparable to in vitro controls (Fig. 6d).

## Discussion

We present the initial steps toward developing an iBAK that will include a cell-containing bioreactor component to recapitulate native renal tubule function. Specifically, in this pilot study, we demonstrate the feasibility of a bioreactor that utilizes SNM to potentially immunoprotect against hyperacute rejection and sustain HREC in vitro and in vivo. We assembled and implanted SNM-containing bioreactor prototypes and confirmed their patency for up to 7 days in pigs that only received antiplatelet agents. Additionally, the bioreactors supported encapsulated xenogeneic HREC in the immunocompetent pigs without the administration of systemic immunosuppression.

We validated our CFD analysis with an intravascular bioreactor that maintained patency despite a lack of therapeutic or prophylactic anticoagulation. Due to protein adsorption on artificial blood-contacting surfaces and subsequent platelet/leukocyte adhesion and complement activation, biomedical device-associated thrombogenicity is a common concern^33^. Formation of thrombi, or blood clots, can lead to failure of the device and serious thromboembolic complications (such as stroke) in the patient. In clinical practice, oral anticoagulation is frequently given to prevent the formation of or treat thrombi in patients who receive intravascular medical devices^34^. However, therapeutic anticoagulation has associated risks of life-threatening bleeding and the formation of hematoma, which can cause patient- and device-related morbidity. Therefore, there is great interest in utilizing antifouling surfaces in blood-contacting devices to reduce thrombus formation. The SNM used in this bioreactor are modified with a PEG coating to reduce cell attachment and protein adsorption. SEM imaging of explanted SNM after direct blood contact confirmed minimal cell and protein attachment. While the pigs did receive antiplatelet agents perioperatively, the lack of thrombi is a significant feat and an important step in demonstrating a safe iBAK.

Additionally, the SNM seemed immunoprotective against hyperacute rejection, successfully allowing the passage of waste, oxygen, and other vital nutrients to maintain cells while preventing the passage of immunologic mediators associated with post-transplant rejection. Benchtop testing demonstrated that the SNM were effective barriers to the pro-inflammatory cytokine TNF-a, and the HREC isolated by SNM maintained viability and confluence. In contrast, the HREC exposed to a supraphysiologic concentration of TNF-a had significantly reduced cell viability with reduced ZO-1 expression. The optimal pore size of the SNM (~10 nm) allows for selective permeability, preventing the passage of large cytokines and even larger immune system components, such as T cells and antibodies. The ability of SNM to provide an immune sanctuary was further corroborated in vivo, where bioreactors implanted in fully immunocompetent pigs sustained human cells. Analysis of the HREC encapsulated within the bioreactor demonstrated high levels of viability, comparable to healthy in vitro control cells. Encapsulated cells were also evaluated with assays measuring NAG and GGT activity at 3 and 7 days after implant. NAG is a lysosomal enzyme that catalyzes the hydrolysis of terminal glucose residues in glycoproteins. It serves as a biomarker of nephrotoxicity and an early warning of renal graft rejection due to its sensitivity to acute ischemic and oxidative stress^35–37^. NAG levels in explanted HREC were slightly lower than those of the in vitro control cells. GGT is a glycoprotein located on the brush border of renal cells associated with glutathione metabolism^38^. Usually upregulated during ischemia, GGT is associated with loss of brush border integrity. In the explanted cells, GGT levels were comparable to those of in vitro control cells.

Cytokine levels of the pigs were also quantified to evaluate the recipient response to implanted xenogeneic cells. Of the 13 common inflammatory biomarkers measured, some were below the limit of the linear range of detection (LLD). A transient increase in expression of some cytokines at 2 days after implant can be attributed to expected postoperative inflammation. By day 7, an adaptive immune response would have already occurred, with corresponding increases in cytokine levels. However, 7 days after implant, all cytokine levels decreased to below intra-operative levels, reflecting a state of minimal inflammation. This is the opposite of the vigorous response that would be expected in a typical xenograft model with an immunocompetent recipient. To parse out specific inflammatory responses triggered by cells, cytokines from bioreactor implants were also compared to implants of similar devices without cells. Cytokine concentrations after the implant of devices without cells were relatively low and did not change markedly on days 2 or 7. Overall, these data suggest a lack of persistent and adaptive immune activation in response to the implanted bioreactors. It is possible that the small number of cells used in this initial proof-of-concept study was insufficient to adequately provoke a recipient’s immune response. While we hypothesize that the SNM prevents the passage of immune system mediators, thus preventing recipient immune activation, further investigation is necessary for future devices that will contain more cells.

Our experimental design was optimized to study cell viability rather than transport or metabolic function. The relatively small number of cells in the bioreactor (1.2 × 10^7^) limited our ability to monitor for evidence of volume transport. Therefore, we measured the gene expression of transporters AQP1 and NHE3 in the encapsulated HREC after explant. AQP1 encodes for an aquaporin, or a molecular water channel protein and a non-selective cation channel^39^. NHE3 is a sodium-hydrogen antiporter that transports Na+ into the cell and pumps H+ out^40^. For the encapsulated cells, NHE3 expression was upregulated roughly twofold relative to the in vitro control. Renal cell function, particularly NHE3 expression, has been reported to increase significantly in biomimetic environments with the application of shear stress^41^. Therefore, it is not entirely unexpected to observe low NHE3 expression in static in vitro control cells. Moreover, it is possible that the immunoprotected milieu within the bioreactor promoted the capacity of the cells to transport Na+ and, indirectly, support volume homeostasis. This suggestion of possible immunoprotection is further supported by the gene expression of 1a Hydroxylase for vitamin D hydroxylation, which was increased by 1.5-fold relative to the static control. Collectively, these data support the hypothesis that SNM can immunoprotect as well as sustain the viability and function of encapsulated HREC.

This report presents the first proof-of-concept of an implantable bioreactor that sustains human renal cells in a porcine model. This study was undertaken for 3 or 7 days to simulate the time in which hyperacute rejection would typically occur^42^. Here we demonstrated the feasibility of a bioreactor with promising features, such as a potential lack of need for immunosuppression or systemic anticoagulation, with no consequence to cell viability or function. Future efforts will focus on increasing both cell numbers and implantation periods with an increased number of animals to establish statistical significance and definitive proof. While the total proximal tubule cell number in a human kidney is 5 × 10^9^ cells^22^, we envision incorporating increased cell numbers beyond our current work to perform some level of clinically meaningful renal function without sacrificing implantability. With the iterative design of the bioreactor housing, SNM porosity, and assembly techniques, we anticipate that up to 1.0 × 10^9^ cells can be accommodated in a device suitable for implantation. This target cell number will allow for higher-level characterization of renal cell functionality, verification of our preliminary findings, and a more robust evaluation of the immune response. Moreover, bioreactors incorporating as many as 1 × 10^9^ cells will enable the evaluation of the therapeutic potential of implanted cells in pigs with reduced renal function. Successful outcomes will position the bioreactor for integration with hemofiltration technology to achieve a single device that provides complete renal replacement therapy.

## Methods

### Ethics

The animal study was approved by the IACUC at Labcorp Early Development Laboratories Inc. (San Carlos, CA). Protocol numbers ICA #1996, ANS #2300 and IAC #2312, ANS #2578.

### Silicon membrane fabrication

Silicon nanopore membranes (SNM) were produced using microelectromechanical systems (MEMS) fabrication technology^27,43,44^. The fabrication process involves the growth of an ultrathin layer (10 nm) of SiO_2_ on a first polysilicon film patterned with vertical-walled grooves. The polysilicon film is deposited using low-pressure chemical vapor deposition on the front side of a 400-µm-thick silicon wafer. A second polysilicon film is then similarly deposited, and the film stack is planarized to achieve vertically oriented columns of 10 nm-wide veins of SiO_2_. The wafer backside is then patterned by deep reactive ion etching to create a thin suspended polysilicon membrane, which is subsequently exposed to hydrofluoric acid to remove the SiO_2_ veins, opening 10 nm-wide pores. The SNM wafer is then diced into 10 × 65 mm chips, forming a rectangular membrane chip with a narrow periphery of solid silicon.

The monodisperse pores in the rectangular membrane are small enough to prevent the passage of immune cells, antibodies, complement-reaction mediators, and loss of albumin. However, they are large enough to accommodate nutrient exchange, permitting cell growth on the basal (back) side.

### Surface modification with polyethylene glycol (PEG) to reduce protein adsorption

Protein fouling of SNM could lead to thrombus formation and occlude pores, limiting the passage of nutrients to the encapsulated HREC. Therefore, the polysilicon surfaces of the SNM were modified with a self-assembled layer of PEG (65994-07-2, Gelest Inc) via liquid deposition^27,45–47^.

### Computational fluid dynamics modeling for blood flow path optimization

The device blood flow conduit was designed to minimize thrombus formation using Ansys Fluent software (ANSYS Inc.). Blood flow path models typically contained approximately 6 million combined hexahedral, prism, and tetrahedral elements. Blood was modeled as a non-Newtonian fluid at 37 °C using the Cross viscosity model^48,49^. Transient solutions were obtained using a time-varying mass flow rate inlet boundary condition derived from animal data^50^. Flow path geometries were modified to eliminate regions of flow recirculation and low wall shear stress (WSS). Based on previous observations of the relationship between WSS and thrombogenicity, a lower bound threshold of 1 Pa (10 dynes/cm^2^) WSS was selected to evaluate the thromboresistance of candidate flow paths^51–53^. The bioreactor blood conduit geometry was modified until simulation results exhibited no flow recirculation or sub-threshold WSS.

### Bioreactor housing fabrication

The device housing was fabricated using USP Class VI Zelux GS polycarbonate (Hayes Manufacturing Services, Sunnyvale, CA). To create the internal blood flow path within a single polycarbonate part, mirror image cavities were CNC milled from two blocks of polycarbonate. The two blocks were carefully aligned and bonded together using thermal fusion to create a single unit. This was followed by external machining of the bonded parts along with chemical vapor polishing to improve surface quality. This machining process allowed for the creation of complex internal geometries and smooth transitions from circular to rectangular blood conduit. Graft connectors were milled from 316L stainless steel (Hayes Manufacturing), and cell inserts were milled from acrylic (Hayes Manufacturing). The gaskets were laser-cut from USP Class VI silicone sheets (McMaster Carr).

### Primary HREC culture

HRECs were obtained from cadaveric kidneys not suitable for transplant from Innovative Biotherapies (HREC-EP092110) (Ann Arbor, MI) via the National Disease Research Interchange. Cells were processed as described by Humes et al.^54^. Cells were cultured at 37 °C, 5% CO_2_ and grown to confluence. The cells were maintained in UltraMDCK medium supplemented with 1 mL/L each of insulin (BW12-749Q, BioWhittaker), transferrin, ethanolamine, and selenium (ITES, BW17-839Z, BioWhittaker), 0.7 μg/L triiodothyronine (709719, Sigma), 50 μg/L epidermal growth factor (EGF, 236-EG, R&D systems), 100 IU/mL penicillin-streptomycin (15070063, Invitrogen), and 10^−7^ M Retinoic Acid (r2625, Sigma) according to supplier instructions.

### Immunoprotective barrier characterization

An SNM was placed in the center of a 24 mm-diameter Transwell® 6-well insert (#3450, Corning, NY) and bonded in place with polydimethylsiloxane (PDMS) (Sylgard 184, Dow Corning). Prior to cell culture, the bonded SNM-Transwell® inserts were sterilized in 70% ethanol for 15 min and then rinsed multiple times with sterile deionized water. The sterilized SNM-Transwell® inserts were coated with mouse collagen type IV (25 μg/mL) (354233, Corning) and rinsed with phosphate-buffered saline (PBS) (UCSF Cell Culture Facility) after 1 h at room temperature. The SNM-Transwell® inserts were placed inside the wells of a standard 6-well tissue culture plate that was similarly coated with mouse collagen type IV. HRECs were seeded on the SNM-Transwell® inserts (“apical compartment”) and on the bottoms of the 6-well plates (“basal compartment”) at a density of 5 × 10^5^ cells/cm^2^. The SNM-Transwell® inserts were maintained in the wells of the 6-well plates, and all cells were cultured for 3–5 days until confluent. To evaluate the effects of cytokine exposure, 300 ng/mL of human tumor necrosis factor- α (TNF-a) (300-01 A, PeproTech) was added to the apical compartment only and then incubated for 6 h. A similar setup was used without cells as a control.

After 6 h of incubation, TNF-a levels were quantified in both the apical and basal compartments of cell-containing and control setups using the Human ELISA kit (BMS223-4, Thermo Fisher Scientific) according to the manufacturer’s instructions. All experiments were completed at least in triplicate.

### Cell characterization after cytokine exposure

After 6 h of exposure to TNF-a, the cell viability, monolayer integrity and cell tight junction expression of both cell compartments was assessed via immunofluorescence.

#### Cell viability

HREC viability was evaluated by fluorescence imaging using the LIVE/DEAD™ viability/cytotoxicity kit (R37601, Invitrogen). Live cells were stained with calcein AM (green color) and dead cells with ethidium homodimer-1 (red color) and immediately imaged with an epifluorescence microscope (Ti-E microscope, Nikon Instruments Inc). ImageJ software was used to quantify cell viability from imaging.

#### Immunofluorescence staining

The monolayer integrity and cell tight junction expression were assessed via immunofluorescence stain for zonula occludens-1 (ZO-1) to visualize cell–cell junctions. Cells were fixed in 4% paraformaldehyde (J61899-AK, Thermo Scientific) at room temperature for 20 min. Cells were then washed 3x with PBS, permeabilized with 0.1% Triton-X100 (UCSF Cell Culture Facility) for 10 min, and blocked in 5% goat serum (ab7481, Abcam) for 30 min. Cells were incubated in Alexa Fluor 488 Conjugated mouse ZO-1 (1:200) (339188, Invitrogen) antibodies in blocking buffer for 1 h at room temperature, then washed 3x with PBS and mounted with the nuclei stain 4′,6-diamidino-2-phenylindole (DAPI) (ab104139, Abcam). Cells were imaged on an epifluorescence microscope (Ti-E microscope, Nikon Instruments Inc.) and processed using ImageJ software.

### Bioreactor cell culture and assembly

Acrylic cell inserts (7 × 1.5 cm, length/width) with six 6 × 6 mm windows cut out were produced using a VLS Laser cutter (Universal Laser Company). The acrylic inserts were permanently affixed to a 0.4 μm polycarbonate Transwell® membrane (#7910, Corning) using epoxy (302-3 M, EPO-TEK). Prior to cell culture, the Transwell®-bonded acrylic inserts (“cell inserts”) were sterilized in 70% ethanol for 15 min and then rinsed with sterile deionized water. The sterilized cell inserts were coated with mouse collagen type IV (25 μg/mL) (354233, Corning) and rinsed with PBS after 1 h of setting time at room temperature. HRECs were seeded on the cell inserts into the recesses from the six windows at a seeding density of 5 × 10^5^ cells/cm^2^. Cells were cultured under static conditions for 3–5 days until adherent and confluent to the inserts. Cell media was changed every 2–3 days.

The devices were assembled while immersed in a 37 °C PBS bath in a sterile cell culture hood to prevent the formation of air bubbles. The SNM, silicone gasket, confluent cell insert, and a second silicone gasket were stacked and fastened in place with stainless steel 316 screws (92185A077, McMaster-Carr). The process was duplicated for both the top and bottom sides of the bioreactor, for a total of four SNM, four cell inserts, and eight silicone gaskets 500 µm thick. Once fully assembled, the device was filled with cell culture media, the inlets and outlets were capped, and the device was placed in a sterile sleeve for implantation.

### Bioreactor implantation

Five healthy female adolescent Yucatan minipigs (*Sus scrofa domestica*) aged 6–8 months and weighing 40–60 kg were selected. Three days prior to bioreactor implantation, the animals were started on aspirin (325 mg by mouth daily) and clopidogrel (75 mg by mouth daily), which continued until surgical retrieval of the device. A study duration of 3 (three pigs) or 7 days (two pigs) was selected to replicate the timing of onset of hyperacute rejection. Initial 3-day proof-of-concept implants were performed subcutaneously in the neck for ease of vascular access and to facilitate postoperative monitoring. Subsequent 7-day studies were performed via a retroperitoneal approach to protect the bioreactor from external trauma and to develop a clinically translatable implantation strategy. Implants were performed under general anesthesia. For the 7-day retroperitoneal implants, a curvilinear incision was made in the right lower quadrant, extending superiorly from the suprapubic space. Dissection was extended through Scarpa’s fascia, and the retroperitoneum was entered through the obliques. The retroperitoneum was dissected free using blunt dissection, and the peritoneum was moved medially and superiorly, revealing the epigastric vessels. These were ligated with silk ties and traced to the iliac artery and iliac vein. For the 3-day neck implants, an incision was made lateral to the right sternocleidomastoid muscle and dissection carried to the internal carotid artery and external jugular vein.

Both vessels were isolated, and 100 units/kg of intravenous heparin was administered prior to vessel occlusion. Ringed 6 mm-diameter polytetrafluoroethylene (PTFE) grafts (Gore Medical) were spatulated and connected in an end-to-side manner to the artery and vein, using 6-0 Prolene suture (Ethicon US, LLC). For retroperitoneal implants, a device pocket was extended bluntly from the pelvic vessels to the inferior pole of the right kidney. During cervical implants, a second incision was made on the dorsolateral surface of the neck to allow a 6 × 10 cm subcutaneous pocket to accommodate the device, while grafts were tunneled from the ventral neck incision. The distal ends of the PTFE arterial and venous grafts were covered by a 2-mm-thick Dragon Skin™ silicone (10 Slow, Smooth-On Inc) sleeve for graft protection, then connected to the inflow and outflow barbs, respectively. Venous and arterial clamps were removed, perfusing the device.

On postoperative day 3 or 7, animals were anesthetized, and an angiogram was performed to assess the patency of the device. Bioreactors were exposed and retrieved prior to euthanizing the animals. Devices were immediately rinsed gently with 1 liter of normal saline to prevent gross clot formation and placed into a normal saline bath for transport from the animal facility to the laboratory. After device disassembly, the SNM were examined by optical and scanning electron microscopy (SEM) for defects and evidence of thrombi or cellular deposits.

### Assessment of recipient immune response to xenograft model

To assess the recipient’s immune response to the presence of human renal cells, samples of the pigs’ venous blood were collected on the day of the implant at the end of the procedure and either 2 or 7 days postoperatively. Plasma was collected and stored in EDTA at −80 °C. As controls, samples of pig plasma were collected at the same time points from other surgeries, where non-cell-containing hemofiltration devices were implanted through the same approach. All samples were run with the Porcine Cytokine 13-Plex Discovery Array (Eve Technologies), a panel of 13 common biomarkers of inflammation, according to the manufacturer’s protocol.

### Scanning electron microscopy

After explant, the SNM were placed in a solution containing 2% glutaraldehyde (16000, Electron Microscopy Sciences), 3% sucrose (Sigma-Aldrich) and 0.1 M of PBS at 4 °C and pH 7.4 for 1 h. The substrates were rinsed with PBS for 30 min at 4 °C and washed with distilled water for 5 min, then dehydrated by placing in 50% ethanol for 15 min while increasing the concentration of ethanol to 100% in increments of 10%. Dehydrated samples were mounted on aluminum stubs, sputter-coated with gold-palladium, and examined using SEM (Ultra55 FEGSEM, ZEISS) at the San Francisco State University Electron Microscopy Facility.

### Characterization of explanted cells

#### Cell viability and monolayer integrity

HREC viability of the cell inserts was imaged and quantified using the LIVE/DEAD™ viability/cytotoxicity kit (R37601, Invitrogen) and ImageJ, as described above. Immunofluorescence staining for ZO-1 was performed to visualize cell–cell junctions, and DAPI to visualize nuclei, as explained above.

#### Cell injury assessment of explanted cells

Cell inserts were incubated in 0.25% Trypsin-EDTA (25200072, Gibco) at 37 °C for 7 min. Cell inserts were gently rinsed with PBS and all cells were collected, resuspended in cold PBS, and spun down to form cell pellets. The cell pellets were resuspended in a cell assay buffer. To quantify cell damage activity of N-acetyl-β-glucosaminidase (NAG) was measured using a colorimetric NAG activity assay (ab204705, Abcam), normalized to total cellular protein. The activity of Gamma-glutamyl transpeptidase (GGT) was measured using a colorimetric r-GT activity assay kit (MAK089, Sigma-Aldrich). Activity levels were normalized to in vitro control cells, as well as the provided standard positive control.

#### Gene expression of functional markers

Quantitative real-time polymerase chain reaction (qPCR) was used to quantify gene expression levels for NHE3, AQP1, and 25-Hydroxyvitamin D3 1-∝-hydroxylase (1a Hydroxylase). Complementary DNA (cDNA) was synthesized by reverse transcription using the SYBR™ Green Fast Advanced Cells-to-CT™ Kit (a35379, Invitrogen). Forward and reverse primers for NHE3 (Hs00903842_m1, Invitrogen), AQP1 (Hs00166067_m1, Invitrogen) and 1a Hydroxylase (Hs01096154_m1, Invitrogen) were utilized. qPCR assays were performed using 20 nM of primer and a 1:10 dilution of cDNA. Expression levels were normalized to the housekeeping gene Glyceraldehyde 3-phosphate dehydrogenase (GAPDH) and to in vitro control cells.

### Statistical methods

Sample pairs were analyzed using Student’s *t*-test. Multiple samples were evaluated with one-way or two-way analysis of variance (ANOVA) followed by Bonferroni and multiple comparison using GraphPad Prism software (San Diego, CA). A *p*-value of <0.05 was accepted as statistically significant for all analyses.

### Reporting summary

Further information on research design is available in the Nature Portfolio Reporting Summary linked to this article.

### Supplementary information

Reporting Summary

### Source data

Source Data

## Data Availability

All relevant data supporting the key findings of this study are available within the article and its Supplementary Information files or from the corresponding author upon reasonable request. Source data are provided with this paper.
