# Supplementary material for: Feasibility of an implantable bioreactor for renal cell therapy using silicon nanopore membranes
Source: Nat Commun. 2023 Aug 29;14:4890. doi: 10.1038/s41467-023-39888-2 (PMC10465514; doi:10.1038/s41467-023-39888-2)
Supplement: Supplementary file 1 — Reporting Summary [file 41467_2023_39888_MOESM1_ESM.pdf]

nature portfolio

Corresponding author(s): Shuvo Roy

Last updated by author(s): Mar 30, 2023

# Reporting Summary

Nature Portfolio wishes to improve the reproducibility of the work that we publish. This form provides structure for consistency and transparency in reporting. For further information on Nature Portfolio policies, see our Editorial Policies and the Editorial Policy Checklist.

## Statistics

For all statistical analyses, confirm that the following items are present in the figure legend, table legend, main text, or Methods section.

| n/a | Confirmed | |
|---|---|---|
| ☐ | ☒ | The exact sample size ($n$) for each experimental group/condition, given as a discrete number and unit of measurement |
| ☐ | ☒ | A statement on whether measurements were taken from distinct samples or whether the same sample was measured repeatedly |
| ☐ | ☒ | The statistical test(s) used AND whether they are one- or two-sided<br>*Only common tests should be described solely by name; describe more complex techniques in the Methods section.* |
| ☒ | ☐ | A description of all covariates tested |
| ☒ | ☐ | A description of any assumptions or corrections, such as tests of normality and adjustment for multiple comparisons |
| ☒ | ☐ | A full description of the statistical parameters including central tendency (e.g. means) or other basic estimates (e.g. regression coefficient) AND variation (e.g. standard deviation) or associated estimates of uncertainty (e.g. confidence intervals) |
| ☒ | ☐ | For null hypothesis testing, the test statistic (e.g. $F$, $t$, $r$) with confidence intervals, effect sizes, degrees of freedom and $P$ value noted<br>*Give P values as exact values whenever suitable.* |
| ☒ | ☐ | For Bayesian analysis, information on the choice of priors and Markov chain Monte Carlo settings |
| ☒ | ☐ | For hierarchical and complex designs, identification of the appropriate level for tests and full reporting of outcomes |
| ☒ | ☐ | Estimates of effect sizes (e.g. Cohen's $d$, Pearson's $r$), indicating how they were calculated |

*Our web collection on statistics for biologists contains articles on many of the points above.*

## Software and code

Policy information about availability of computer code

| | |
|---|---|
| Data collection | No software was used |
| Data analysis | GraphpadPrism, Microsoft Excel, ANSYS Fluent, ImageJ |

For manuscripts utilizing custom algorithms or software that are central to the research but not yet described in published literature, software must be made available to editors and reviewers. We strongly encourage code deposition in a community repository (e.g. GitHub). See the Nature Portfolio guidelines for submitting code & software for further information.

## Data

Policy information about availability of data

All manuscripts must include a data availability statement. This statement should provide the following information, where applicable:

- Accession codes, unique identifiers, or web links for publicly available datasets
- A description of any restrictions on data availability
- For clinical datasets or third party data, please ensure that the statement adheres to our policy

All data generated in this study are provided in the Supplementary Information/Source Data file. Source data are provided with the paper.

## Human research participants

Policy information about studies involving human research participants and Sex and Gender in Research.

| | |
|---|---|
| Reporting on sex and gender | N/A |
| Population characteristics | N/A |
| Recruitment | N/A |
| Ethics oversight | N/A |

Note that full information on the approval of the study protocol must also be provided in the manuscript.

# Field-specific reporting

Please select the one below that is the best fit for your research. If you are not sure, read the appropriate sections before making your selection.

☒ Life sciences ☐ Behavioural & social sciences ☐ Ecological, evolutionary & environmental sciences

For a reference copy of the document with all sections, see nature.com/documents/nr-reporting-summary-flat.pdf

# Life sciences study design

All studies must disclose on these points even when the disclosure is negative.

| | |
|---|---|
| Sample size | No sample size calculation was specifically performed. Sample sizes were chosen based on review of published literature where similar types of experiments were conducted. |
| Data exclusions | No data were excluded. |
| Replication | Protocols were validated with controls. Replication of experimental findings were successful except for some animal experiments due to budget and pandemic related constraints. Total numbers of each experiment performed are stated in figure legends |
| Randomization | Randomly allocated. |
| Blinding | Blinding was not possible because control samples were external (cell incubators) while experimental samples were implanted. |

# Reporting for specific materials, systems and methods

We require information from authors about some types of materials, experimental systems and methods used in many studies. Here, indicate whether each material, system or method listed is relevant to your study. If you are not sure if a list item applies to your research, read the appropriate section before selecting a response.

### Materials & experimental systems

| n/a | Involved in the study | |
|---|---|---|
| ☐ | ☒ | Antibodies |
| ☐ | ☒ | Eukaryotic cell lines |
| ☒ | ☐ | Palaeontology and archaeology |
| ☐ | ☒ | Animals and other organisms |
| ☒ | ☐ | Clinical data |
| ☒ | ☐ | Dual use research of concern |

### Methods

| n/a | Involved in the study | |
|---|---|---|
| ☒ | ☐ | ChIP-seq |
| ☒ | ☐ | Flow cytometry |
| ☒ | ☐ | MRI-based neuroimaging |

## Antibodies

| | |
|---|---|
| Antibodies used | Alexa Fluor 488 Conjugated mouse ZO-1 (1:200) (339188, Invitrogen) |
| Validation | The antibodies were purchased from a commercially credible source and upon reviewing the manufacturer-provided Quality Certificates |

# Eukaryotic cell lines

Policy information about cell lines and Sex and Gender in Research

| | |
|---|---|
| Cell line source(s) | HREC were obtained from human cadaveric kidneys not suitable for transplant from Innovative Biotherapies (HREC-EP092110) (Ann Arbor, MI) |
| Authentication | Cells were authenticated from Innovative Biotherapies |
| Mycoplasma contamination | Cells were tested negative for Mycoplasma by Innovative Biotherapies |
| Commonly misidentified lines (See ICLAC register) | N/A |

# Animals and other research organisms

Policy information about studies involving animals; ARRIVE guidelines recommended for reporting animal research, and Sex and Gender in Research

| | |
|---|---|
| Laboratory animals | Healthy adolescent Yucatan minipigs aged 6-8 months and weighing 40-60 kg. |
| Wild animals | No wild animals were used. |
| Reporting on sex | Findings are applicable to both sexes as there are no relevant physiologic differences that would alter immune or coagulation pathways. However, for operative consideration, female pigs only were preferentially used. The male pig genitalia interfere with the lower abdominal incision. |
| Field-collected samples | No field-collected samples. |
| Ethics oversight | Labcorp Early Development Laboratories Inc. (San Carlos, CA) |

Note that full information on the approval of the study protocol must also be provided in the manuscript.
